# State Feedback and Synergetic controllers for tuberculosis in infected population

**DOI:** 10.1049/syb2.12013

**Published:** 2021-03-30

**Authors:** Muhammad Bilal, Iftikhar Ahmad, Sheraz Ahmad Babar, Khurram Shahzad

**Affiliations:** ^1^ School of Electrical Engineering and Computer Science (SEECS) National University of Sciences and Technology (NUST) Islamabad Pakistan

## Abstract

Tuberculosis (TB) is a contagious disease which can easily be disseminated in a society. A five state Susceptible, exposed, infected, recovered and resistant (SEIRs) epidemiological mathematical model of TB has been considered along with two non‐linear controllers: State Feedback (SFB) and Synergetic controllers have been designed for the control and prevention of the TB in a population. Using the proposed controllers, the infected individuals have been reduced/controlled via treatment, and susceptible individuals have been prevented from the disease via vaccination. A mathematical analysis has been carried out to prove the asymptotic stability of proposed controllers by invoking the Lyapunov control theory. Simulation results using MATLAB/Simulink manifest that the non‐linear controllers show fast convergence of the system states to their respective desired levels. Comparison shows that proposed SFB controller performs better than Synergetic controller in terms of convergence time, steady state error and oscillations.

## 1 INTRODUCTION

**FIGURE 1:**
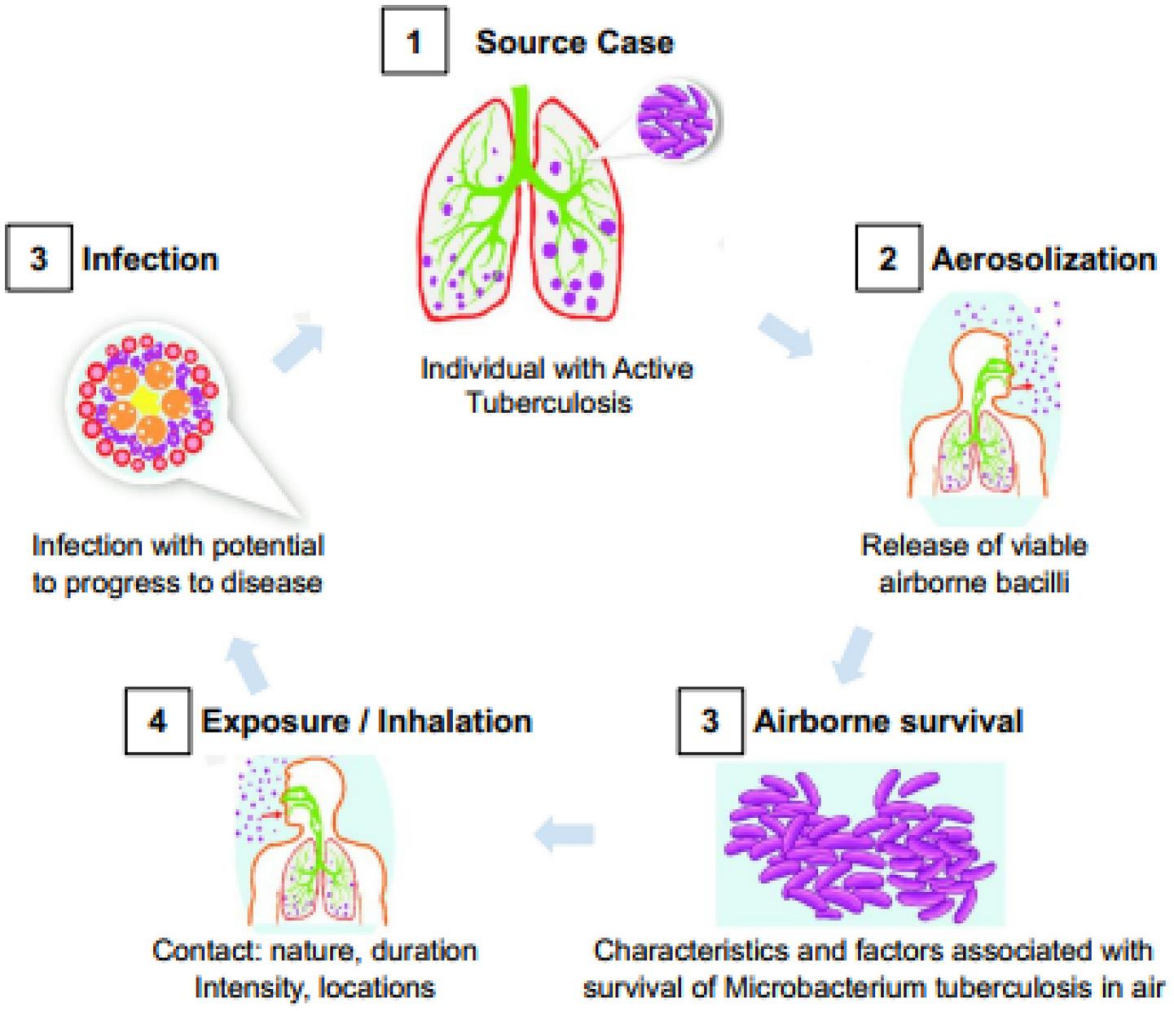
Transmission of tuberculosis in population [2]

Tuberculosis (TB), having two major types, MDR tuberculosis and XDR tuberculosis, is caused by a bacterium called *mycobacterium*. It is one of the top 10 causes of death across the world [1], which affects mainly the human lungs apart from other parts like brain, bones, kidney and spine. It is a transferable disease that can spread over a population. Figure 1 shows that when an infected individual exhales, *mycobacterium* is transferred to the air that can affect the healthy individuals in the surrounding. The other reasons of TB are bad living conditions, malnourishment, smoking, and so forth.

Microbacterium Tuberculosis (Mtb) patients who show resistant to anti‐TB drugs, Isoniazed and Rifampicin, are termed as MDR patients and who show resistant to any injectable anti‐TB drug are termed as XDR. These types of patients do not respond to 6 or 9 months’ treatment. They may take about 2 years of treatment with high toxic anti‐TB medicines to fully recover. These types of Mtb patients are real threat to control and prevent the TB from its spread [2]. Individuals who are HIV positive and infected from TB have 20%–40% more chances to develop active TB which is a leading cause of death HIV positive patients [2].

According to the WHO annual report 2019, globally 1.7 billion people are infected with Mtb and approximately 10 million people suffer from TB every year. About 50–500 people per million population are infected across the world. The male‐female ratio of TB is 2:1. It can affect anybody but is more dangerous for the adults. Developing countries are highly burdened from TB because of poverty, bad living conditions, unavailability of treatment facilities and malnourishment.

The spread of TB can be curtailed by timely diagnosis, treatment, improvement of the health facilities and introducing health conscious activities in the society [1]. Early diagnosis of the disease decreases both social and medical impacts. Surveillance of TB can be done by using Google trends [3] and by observing its counter medication [4]. Spread of TB can also be controlled by giving health education to the society. Effect of different health education methods on secondary and primary school students in northern province of Jiangsu has been discussed in [5].

Bio‐mathematics has played a very important role in the development of the mathematical models of epidemic diseases including TB. In previous research works, several mathematical models of TB have been developed. First of all the stability of the model is examined, then a preventive control in the form of vaccination and treatment of infected class is established and an objective function is defined by researchers. Later, an optimal control law for prevention and control of the infectious TB was defined [6, 7]. Discrete TB model with two different infectious compartments has been discussed in [8, 9]. Stability analysis, bifurcation of TB model and complex system modelling of TB in Nigeria have been discussed in [10, 11]. Computer modelling of sensitive type *mycobacterium* TB and modelling using regression analysis have also been studied [12, 13]. Influence of multiple reinfections in TB dynamics is discussed in [14]. Stability analysis has been discussed for five states TB model in [15]. Optimal control technique has been applied on four and five states non‐linear mathematical models of tuberculosis using Pontryagin's maximum principle [16]. A dynamic behaviour of four states of TB transmission and an optimal controller for its treatment has been discussed in [17].

Synergetic control technique takes into account a macro‐variable whose number depends on the number of inputs. It contains the errors of the states which we want to track [18]. It has been applied for tracking of infected cells during anti‐viral therapy [19], to control the growth of cancer cells [20], on systems of non‐linear equations [21], magnetic levitation system [22], to minimize HIV concentration in blood plasma [23] and for stabilizing the medium voltage microgrids [24]. In State Feedback controller design, the output tracks the desired reference signal asymptotically if the reference signal and its derivative are bounded [].

**FIGURE 2:**
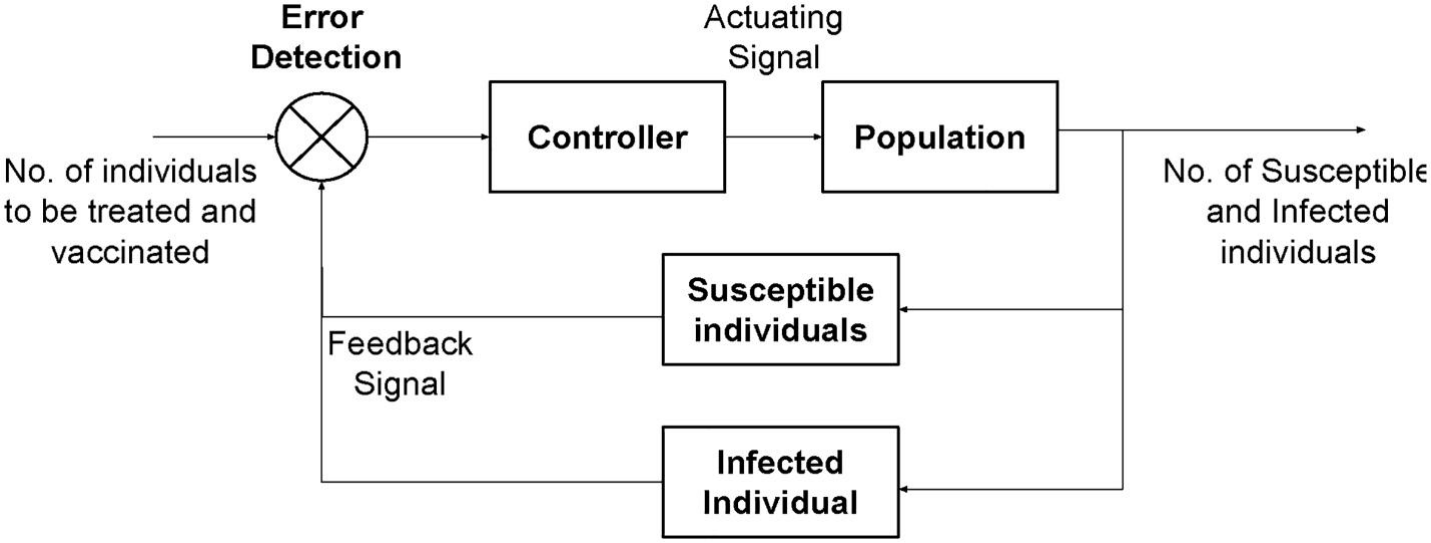
Schematic diagram for close loop control system

In this research work, an updated mathematical model of the TB has been considered to design two non‐linear controllers for preventing the spread of TB and reducing the infected individuals by giving them treatment and vaccination on time. These non‐linear controllers are Synergetic and State Feedback (SFB) controllers which have been designed for the treatment and vaccination of infected population.Schematic diagram for the proposed close loop control system has been shown in Figure 2. The rest of the article is organized as follows; Section 2 describes the non‐linear mathematical model of TB considered for this research. Section 2.2 details the problem statement, and Section 3 describes the design of the proposed non‐linear controllers. Simulation results have been presented in Section 4, where the comparison of the proposed controllers has been made and finally the article has been concluded in Section 4.

## 2 NON‐LINEAR TUBERCULOSIS MODEL

TB is an airborne disease that can spread from person to person via aerosolization to the individuals and through air.

### 2.1 SEIR tuberculosis model

There are a number of mathematical models for the transmission of TB. SIR model [28] incorporates the three state variables: susceptible, infected and recovered. Latest model of the transmission of the Mtb is a five state SEIR model [29] which describes the transmission of the Mtb in a human host taking into account the effect of the MDR and XDR without making the model complicated. In this model, human population is classified into five classes; Infected (*x*_1_), Susceptible (*x*_2_), Exposed (*x*_3_), Recovered (*x*_4_) and Resistant (*x*_5_). Size of the human population *N* can be written as

(1) $$N=x_{1}+x_{2}+x_{3}+x_{4}+x_{5}$$

where the recruitment to the susceptible class is taken by birth rate (*λ*). Size of each class varies due to natural death rate and rates at which the individuals become susceptible, exposed, infected, recovered and resistant. The model is given below.

(2) $${\overset{\cdot }{x}}_{1}=\epsilon x_{2}-\left(\mu +\alpha +\gamma +\sigma \right)x_{1}-u_{1}$$

(3) $${\overset{\cdot }{x}}_{2}=\beta x_{2}x_{3}-\left(\mu +\epsilon \right)x_{2}$$

(4) $${\overset{\cdot }{x}}_{3}=\lambda N-\beta x_{1}x_{3}-\mu x_{3}+\rho x_{4}-u_{2}$$

(5) $${\overset{\cdot }{x}}_{4}=\gamma x_{1}-\left(\mu +\rho \right)x_{4}+\left.\delta x_{5}\right)$$

(6) $${\overset{\cdot }{x}}_{5}=\sigma x_{4}\left(\mu +\alpha_{1}+\delta \right)x_{5}$$

Parameters that are used in the model are described as recruitment by births (*λ*), force of infection (*β*), human death rate (*μ*), active TB disease induction rate (*α*), MDR TB disease induction rate (*α*_1_), humans recovery rate from MDR TB (*δ*), rate at which exposed become infectious (*ϵ*), rate at which infected becomes resistant (*σ*) and rate at which recovered becomes susceptible (*ρ*).

### 2.2 Problem statement

There are number of optimal control strategies for the prevention and control of TB, but it still is one of the leading causes of the death worldwide. There is no non‐linear controller purposed for the prevention and control of TB so far in the literature. This model is nonlinear due to presence of terms *x*_2_ *x*_3_ and *x*_1_ *x*_3_ in Equations (3) and (4), respectively. Therefor designing a non‐linear controller would be a good option to cater for the spread of TB, as non‐linear controllers usually show better convergence, lesser steady state error and negligible oscillations and undershoots/overshoots.

## 3 NON‐LINEAR CONTROLLERS DESIGN FOR SEIR TB MODEL

We have considered SEIR TB model given by Equations ((2), (3), (4), (5), (6))–((2), (3), (4), (5), (6)) in order to design the controllers. Two non‐linear controllers, Synergetic and State Feedback controller, are to be designed for treatment and vaccination of infected population. The control inputs *u*_1_ and *u*_2_ give the number of infected and susceptible individuals for the treatment and vaccination respectively.

### 3.1 Synergetic controller design

Synergetic controller is to be designed for the system to track some state of the system to its desired level. Synergetic control technique will be used to design the control input *u*_1_ and *u*_2_. We have taken two macro‐variables, since the number of input variables are two, defined as

(7) $$\sigma_{1}=c_{1}e_{1}+c_{2}e_{3}+c_{4}e_{4}+c_{5}e_{5}$$

and

(8) $$\sigma_{2}=c_{2}e_{2}+c_{3}e_{3}+c_{4}e_{4}+c_{5}e_{5}$$

The error of each state is defined below which is the difference between actual value and reference value of that state.

(9) $$\begin{array}{cc} e_{1} & =x_{1}-x_{1ref}\\ e_{2} & =x_{2}-x_{2ref}\\ e_{3} & =x_{3}-x_{3ref}\\ e_{4} & =x_{4}-x_{4ref}\\ e_{5} & =x_{5}-x_{5ref} \end{array}$$

All the states would track the desired value if the errors converge to zero respectively. Taking the time derivative of Equation (10), we have

(10) $$\begin{array}{cc} {\overset{\cdot }{e}}_{1} & ={\overset{\cdot }{x}}_{1}-{\overset{\cdot }{x}}_{1ref}\\ {\overset{\cdot }{e}}_{2} & ={\overset{\cdot }{x}}_{2}-{\overset{\cdot }{x}}_{2ref}\\ {\overset{\cdot }{e}}_{3} & ={\overset{\cdot }{x}}_{3}-{\overset{\cdot }{x}}_{3ref}\\ {\overset{\cdot }{e}}_{4} & ={\overset{\cdot }{x}}_{4}-{\overset{\cdot }{x}}_{4ref}\\ {\overset{\cdot }{e}}_{5} & ={\overset{\cdot }{x}}_{5}-{\overset{\cdot }{x}}_{5ref} \end{array}$$

Since reference value of each state is constant, so their time derivatives will be zero, we get

(11) $$\begin{array}{cc} {\overset{\cdot }{e}}_{1} & ={\overset{\cdot }{x}}_{1}\\ {\overset{\cdot }{e}}_{2} & ={\overset{\cdot }{x}}_{2}\\ {\overset{\cdot }{e}}_{3} & ={\overset{\cdot }{x}}_{3}\\ {\overset{\cdot }{e}}_{4} & ={\overset{\cdot }{x}}_{4}\\ {\overset{\cdot }{e}}_{5} & ={\overset{\cdot }{x}}_{5} \end{array}$$

Taking time derivative of Equations (7) and (8), gives

(12) $$\begin{array}{ll} {\overset{\cdot }{\sigma }}_{1} & =c_{1}{\overset{\cdot }{e}}_{1}+c_{2}{\overset{\cdot }{e}}_{2}+c_{4}{\overset{\cdot }{e}}_{4}+c_{5}{\overset{\cdot }{e}}_{5} \end{array}$$

(13) $$\begin{array}{ll} & =c_{1}{\overset{\cdot }{x}}_{1}+c_{2}{\overset{\cdot }{x}}_{2}+c_{2}{\overset{\cdot }{x}}_{2}+c_{5}{\overset{\cdot }{x}}_{5} \end{array}$$

(14) $$\begin{array}{ll} {\overset{\cdot }{\sigma }}_{2} & =c_{2}{\overset{\cdot }{e}}_{2}+c_{3}{\overset{\cdot }{e}}_{3}+c_{4}{\overset{\cdot }{e}}_{4}+c_{5}{\overset{\cdot }{e}}_{5} \end{array}$$

(15) $$\begin{array}{ll} & =c_{2}{\overset{\cdot }{x}}_{2}+c_{3}{\overset{\cdot }{x}}_{3}+c_{4}{\overset{\cdot }{x}}_{4}+c_{5}{\overset{\cdot }{x}}_{5} \end{array}$$

The macro‐variables σ_1_ and σ_2_ are supposed to satisfy the dynamic evaluation presented by the following equation

(16) $$T\overset{\cdot }{\sigma }+\sigma =0$$

where *T* represents the convergence rate of σ_1_ and is a positive constant. Putting down the values of σ_1_ and $\overset{\cdot }{\sigma_{1}}$ from Equations (7) and (13) respectively and solving for *u*_1_, we get

(17) $$\begin{array}{cc} u_{1}= & \frac{1}{Tc_{1}}\left(-Tc_{2}\overset{\cdot }{x_{2}}-Tc_{4}\overset{\cdot }{x_{4}}-Tc_{5}\overset{\cdot }{x_{5}}-\sigma_{1}+T\epsilon x_{2}\right.\\ & \left.\;-T\left(\mu +\alpha +\gamma +\sigma \right)x_{1}\right). \end{array}$$

Now, putting down the value of σ_2_ and $\overset{\cdot }{\sigma_{2}}$ from Equations (8) and (15) respectively and solving for *u*_2_, we get

(18) $$\begin{array}{cc} u_{2}= & \frac{1}{rTc_{3}x_{4}}\left(-Tc_{3}\lambda N-\beta x_{1}x_{3}Tc_{3}-\mu x_{3}Tc_{3}-\sigma -Tc_{4}\overset{\cdot }{x_{4}}\right.\\ & \left.\;-Tc_{5}\overset{\cdot }{x_{5}}\right). \end{array}$$

The control input *u*_1_ and *u*_2_ in Equations (17) and (18) are the required controls obtained through the Synergetic control technique which gives the number of infected and susceptible individuals to be treated and vaccinated, respectively. To prove asymptotic stability of Equation (16), we consider the Lyapunov candidate function as

(19) $$V_{3}=\frac{1}{2}\sigma^{2}$$

Taking the time derivative of Equation (19), we get

(20) $${\overset{\cdot }{V}}_{3}=\sigma \overset{\cdot }{\sigma }$$

Putting down the value of $\overset{\cdot }{\sigma }$ from Equation (16), we get

(21) $${\overset{\cdot }{V}}_{3}=\frac{-\sigma^{2}}{T}=\frac{-2}{T}\frac{1}{2}\sigma^{2}$$

Using Equation (19), we can write

(22) $${\overset{\cdot }{V}}_{3}=\frac{-2}{T}V_{3}$$

When *t* is zero *V*_3_ becomes equal to *V*_*o*_ which is its initial value.

(23) $$V_{3}=V_{o}e^{\frac{-2}{T}t}.$$

Hence the dynamical system is exponentially stable using Lyapunov theory.

### 3.2 State Feedback controller design

In order to design the State Feedback controller, we take *x*_1_ as output of the system that is

(24) $$y_{1}=x_{1}=e_{1}+x_{1ref}$$

Taking the time derivative of Equation (24), we have

(25) $$\overset{\cdot }{y_{1}}={\overset{\cdot }{x}}_{1}$$

The state *x*_1_ will track the desired value if the error *e*_1_ will converge to zero. Therefore, taking time derivative of Equation (24) and putting down the value of $\overset{\cdot }{x_{1}}$ from Equation (2), we can write

(26) $${\overset{\cdot }{e}}_{1}=\epsilon x_{2}-\left(\mu +\alpha +\gamma +\sigma \right)x_{1}-u_{1}$$

Error *e*_1_ will converge to zero if Lyapunov candidate function of error *e*_1_ given by Equation (26) is negative definite. For this purpose, we keep

(27) $$\epsilon x_{2}-\left(\mu +\alpha +\gamma +\sigma \right)x_{1}-u_{1}=-F_{1}e_{1}$$

where *F*_1_ is positive constant. Equation (26) becomes

(28) $${\overset{\cdot }{e}}_{1}=-F_{1}e_{1}$$

Solving Equation (27) for *u*_1_, we have

(29) $$u_{1}=\left(\mu +\alpha +\gamma +\sigma \right)x_{1}-\epsilon x_{2}+F_{1}e_{1}$$

The control input *u*_1_ in Equation (29) is the required one obtained through State Feedback control technique which gives the number of infected individuals to be treated. In similar way, we can design *u*_2_ by choosing *x*_3_ as output

(30) $$y_{2}=x_{3}$$

Taking time derivative of the Equation (30), we have

(31) $$\overset{\cdot }{y_{2}}=\overset{\cdot }{x_{3}}$$

Taking time derivative of *e*_3_ given by Equation (5), we have

(32) $${\overset{\cdot }{e}}_{3}={\overset{\cdot }{x}}_{3}$$

Using the value of ${\overset{\cdot }{x}}_{3}$ given by Equation (3), we have

(33) $${\overset{\cdot }{e}}_{3}=\lambda N-\beta x_{3}x_{1}-\mu x_{3}+\rho x_{4}-u_{2}.$$

Error *e*_3_ given by Equation (5) will converge to zero if Lyapunov candidate function of error *e*_3_ given by Equation (33) is negative definite. For this purpose, we keep

(34) $$\lambda N-\beta x_{3}x_{1}-\mu x_{3}+\rho x_{4}-u_{2}=-F_{2}e_{3}$$

Equation (33) becomes

(35) $${\overset{\cdot }{e}}_{3}=-F_{2}e_{3}$$

Solving Equation (34) for *u*_2_, we have

(36) $$u_{2}=\lambda N-\beta x_{3}x_{1}-\mu x_{3}+\rho x_{4}+F_{3}e_{3}$$

The control input *u*_2_ in Equation (36) is the required control obtained through the State Feedback control technique which gives the number of susceptible individuals to be vaccinated.

## 4 SIMULATION RESULTS

In these simulations, we have considered the SIER model given by the Equations (2), (3), (4), (5), (6) and simulated the proposed controllers given by the Equations (17), (18), (29) and (36) in MATLAB/Simulink. In the graphs, time (year) is taken along the *x*‐axis and number of individuals to be treated/vaccinated are taken along *y*‐axis. Reference value of infected and susceptible classes is taken to be zero. Description of the other parameters and their values used for these simulations are given by the Table 1.

**TABLE 1:** Parameters and their values

Sr.#	Parameters	Values
1	Transmission rate (*β*)	0.35/year
2	Infection rate (*ϵ*)	0.25/year
3	Disease induced rate (*α*)	0.01/year
4	Recovery rate due to prompt disease(*γ*)	0.5/year
5	Resistance disease induced death rate (α_1_)	0.0575/year
6	Resistance rate to treatment (*σ*)	0.470104/year
7	Recovery rate after second line of treatment (*δ*)	0.1106456/year
8	Rate of loss of immunity (*I*_*b*_)	0.05/year
9	Natural mortality (*μ*)	0.019896/year

### 4.1 For infected class

**FIGURE 3:**
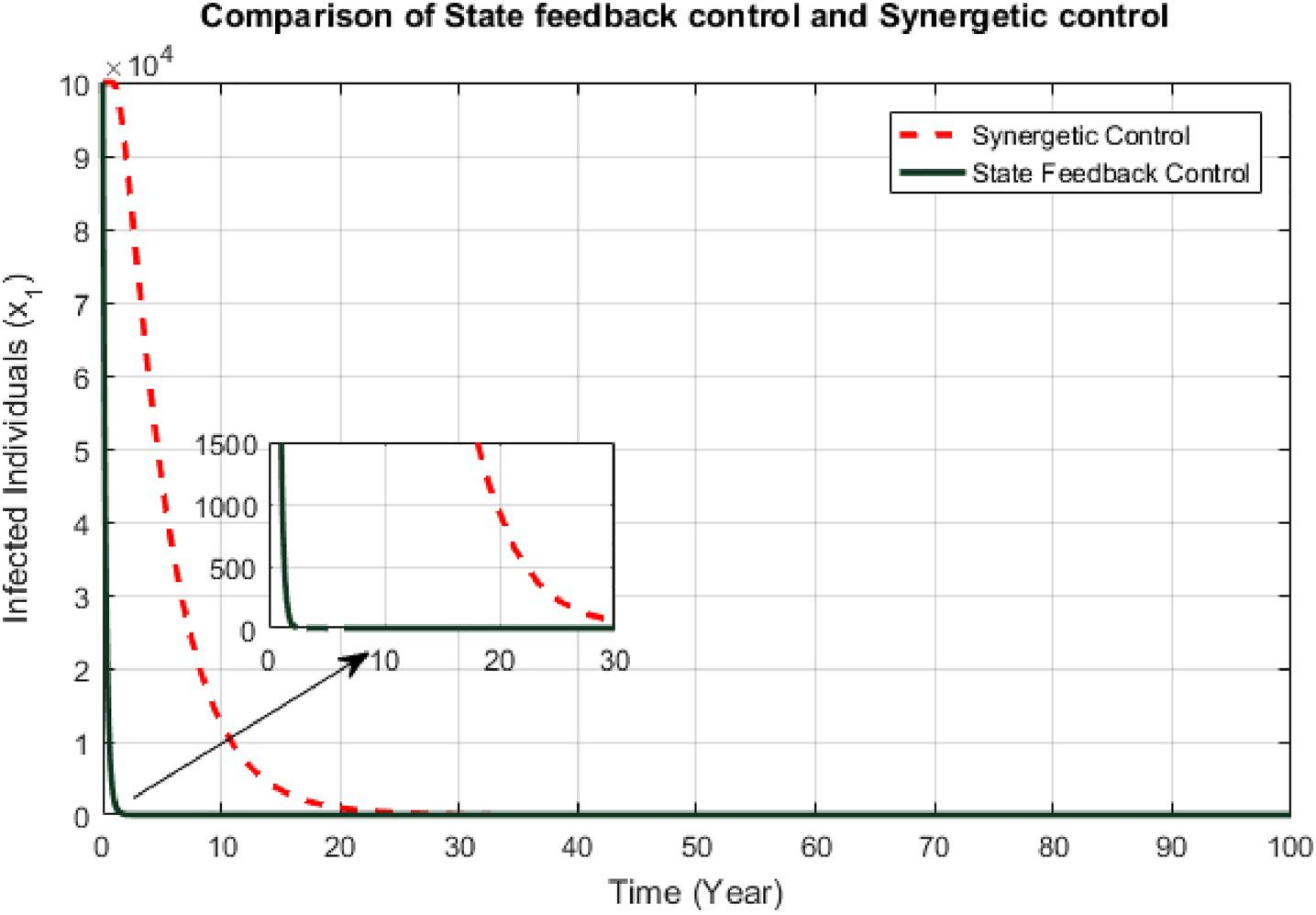
Comparison of infected individuals

Responses of the proposed controllers for the infected class of people have been shown in the Figure 3. It has been observed that convergence time for the SFB controller and Synergistic controller are 1.5  and 20 years respectively and there is no steady state error and oscillations shown by any of the proposed controller.

### 4.2 For susceptible class

**FIGURE 4:**
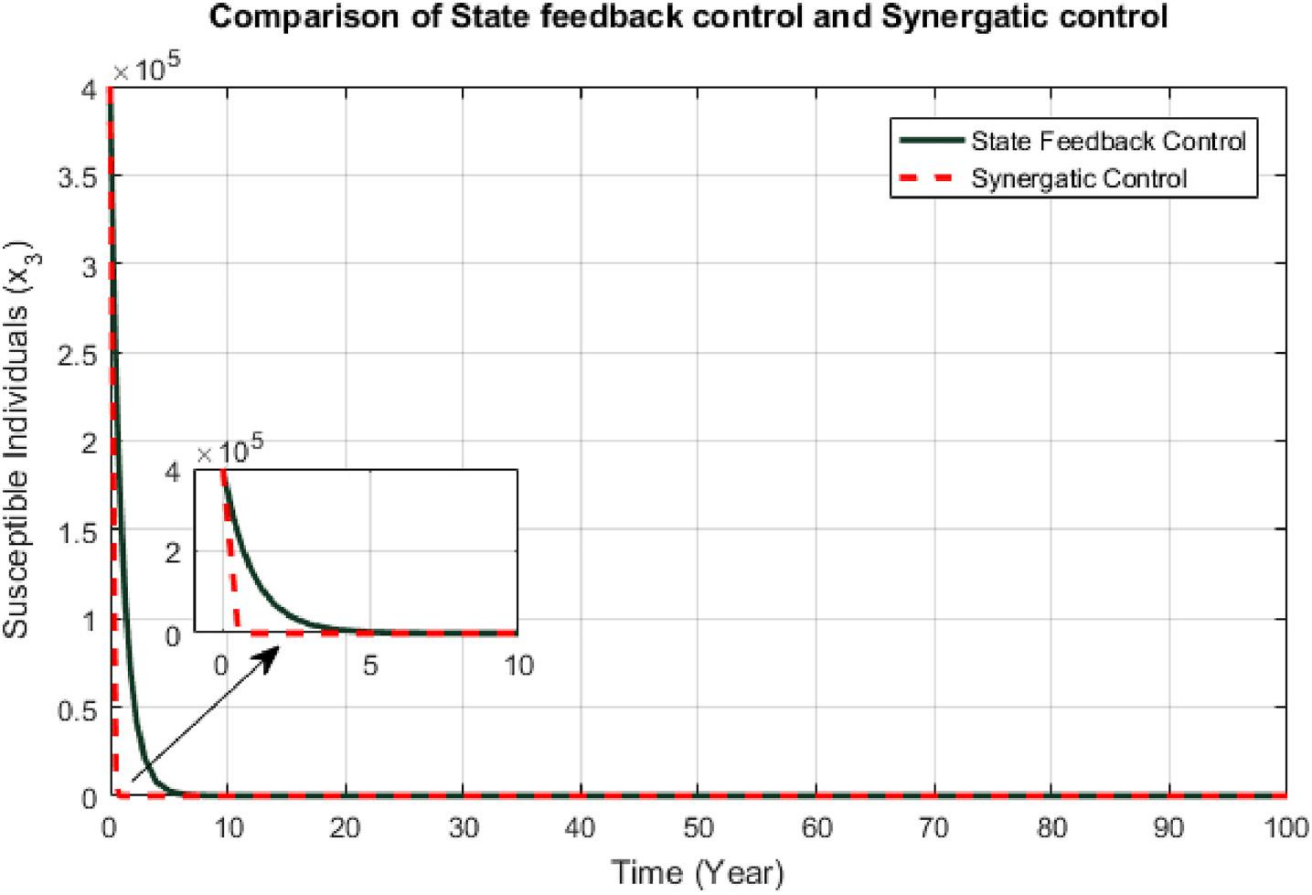
Comparison of susceptible individuals

Responses of the proposed controllers for susceptible class have been shown in Figure 4. The convergence time of the SFB controller and Synergetic controller are 3 years and 1 year, respectively.

### 4.3 Control signal *u*_1_ and *u*_2_ of proposed controllers

**FIGURE 5:**
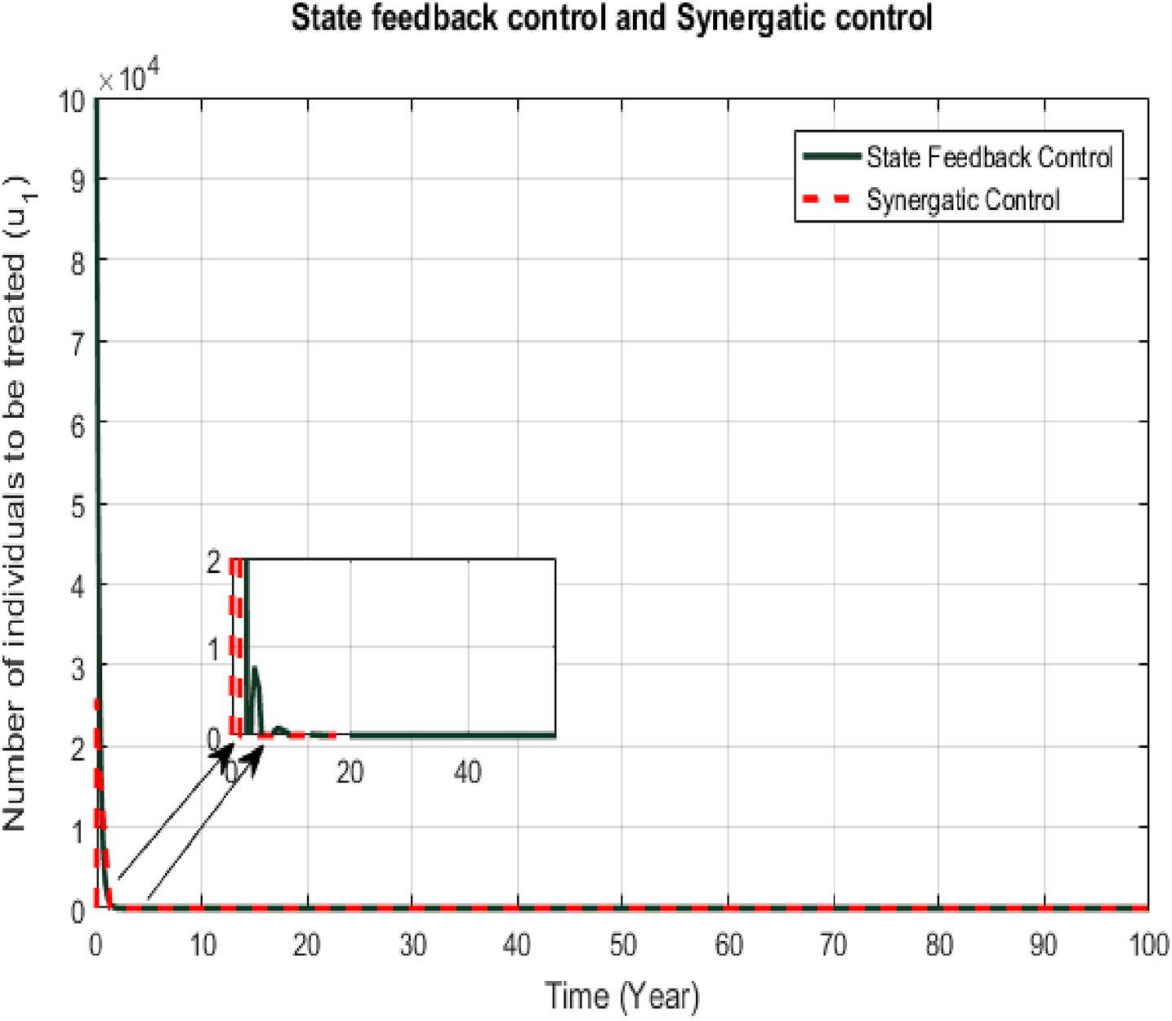
Comparison of *u*_1_

**FIGURE 6:**
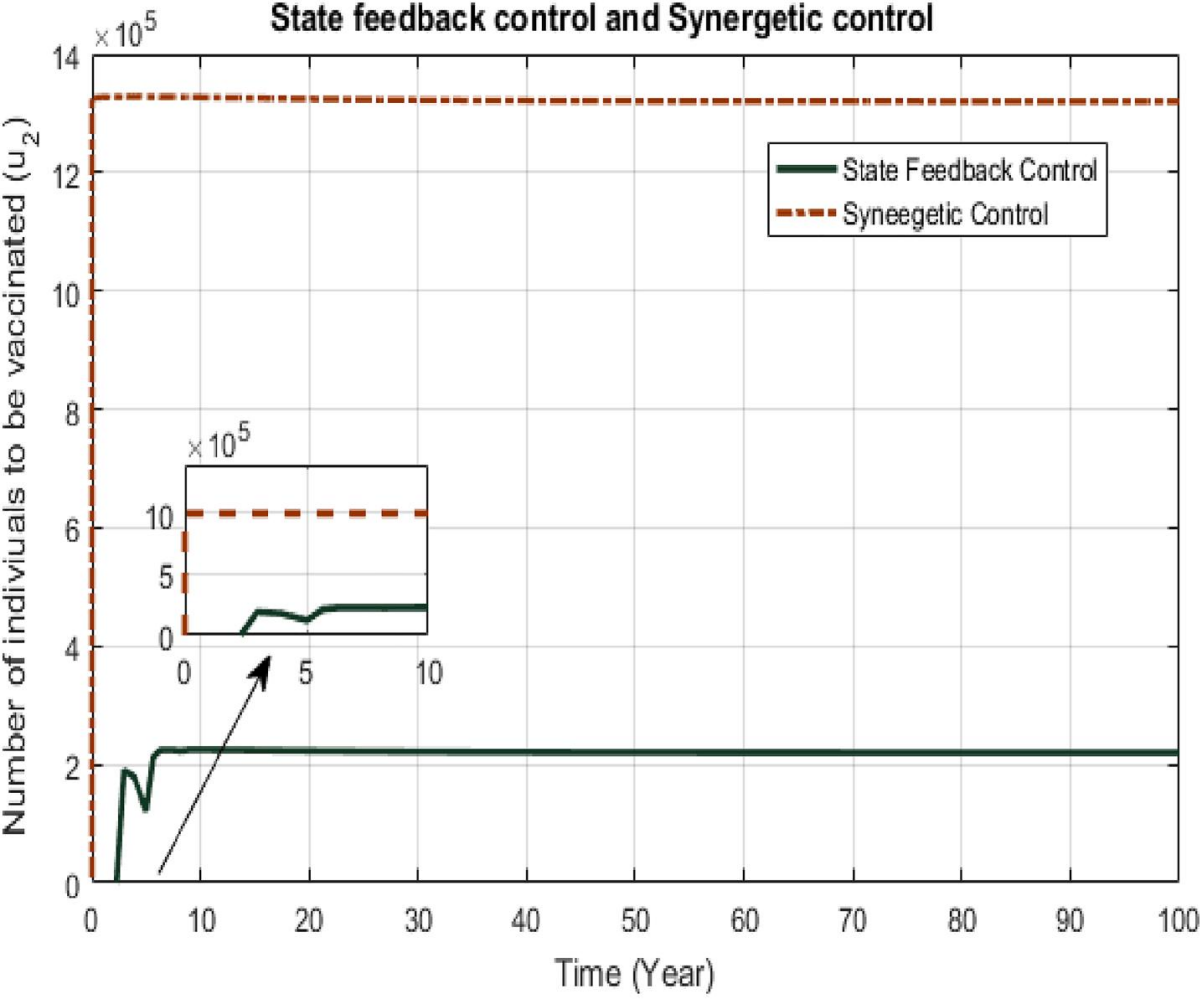
Comparison of *u*_2_

The two control signals from proposed controllers given by Equations (17) and (29) and Equations (18) and (36) are shown in Figures 5 and 6, respectively. The control input *u*_1_ is the signal for the treatment of the infected class which for SFB tracks infected class to zero after 1 year. The control input *u*_1_ of the Synergetic controller tracks the infected class to zero after 1.5. The area under the curve *u*_1_ gives the total number of infected individuals to be given treatment for the cure of TB. The control input *u*_2_ is the vaccination of the susceptible class. As vaccination of TB is the continuous process, each control signal from proposed controller depicts continuous process of vaccination, but with different number of individuals to be vaccinated. The area under the curve *u*_2_ gives the total number of susceptible individuals to be given vaccination.Comparison of two non‐linear controllers: SFB and Synergetic controllers for infected individuals (*x*_1_) and susceptible individuals (*x*_3_) in terms of convergence time, steady state error (SSE) is given in the Table 2.

**TABLE 2:** Example table

Controller	Convergence (Year)	SSE (*x*_1_, *x*_3_)
State Feedback	1	No, No
Synergetic	15	No, No

**FIGURE 7:**
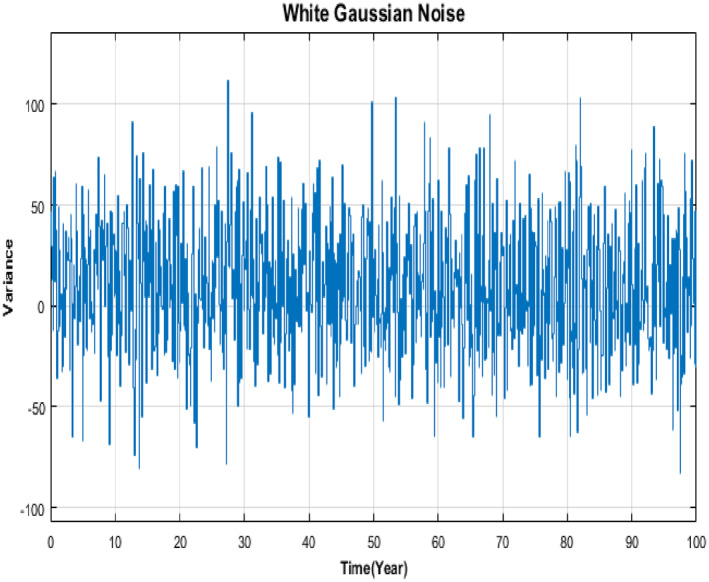
White Gaussian noise

Controller's responses have also been checked for uncertainties or disturbances in the system. The disturbance can be in the form of increase or decrease in number of infected individuals due to migration to the infected population from nearby areas or due to the migration of infected individuals from infected population to other areas respectively. For simulation purpose, this type of disturbance is taken as Gaussian noise with mean 10 and variance 100 as shown in Figure 7.

**FIGURE 8:**
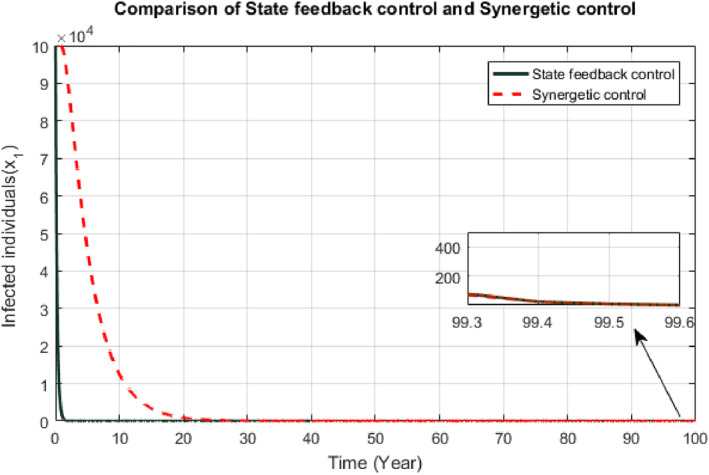
Comparison of infected individuals due to effect of disturbance

**FIGURE 9:**
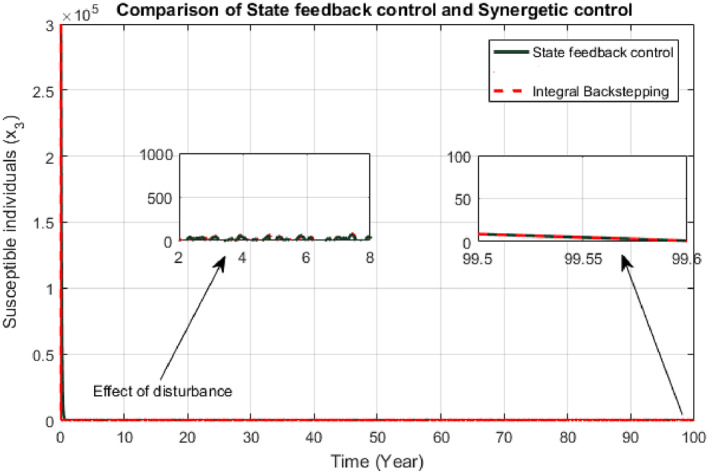
Comparison of susceptible individuals due to effect of disturbance

Figures 8 and 9 exhibit responses of SFB and Synergetic controllers due to this disturbance. Both controllers respond well to the disturbance. When there is increase in the number of infected and susceptible individuals, the response of each controller says that higher number of infected and susceptible individuals would be given treatment and vaccination respectively. SFB controller is not much affected by the disturbance as it shows similar convergence time with negligible oscillations. Convergence time of Synergetic controller is also similar as before, but it takes more time to converge as compared to State Feedback controller.

## 5 CONCLUSION

In this research work, a community‐based five state mathematical model of TB named as SEIR epidemiological, has been considered. This model is unique in the sense that it includes all the states including infected, susceptible, exposed, recovered and resistant classes. SFB and Synergetic controllers have been designed for the prevention and control of this viral disease. Asymptotic stability of the system has been proved using Lyapunov theory. The simulations for the proposed controllers have been performed in MATLAB/Simulink. From the graphs, it is clear that the SFB controller shows good behaviour in terms of the convergence time, steady state error and oscillations as compared to the proposed Synergetic controller.
